# Phenotypic and Genotypic Antimicrobial Resistance Profiles of *Campylobacter jejuni* Isolated from Cattle, Sheep, and Free-Range Poultry Faeces

**DOI:** 10.1155/2009/456573

**Published:** 2010-03-08

**Authors:** Beatriz Oporto, Ramón A. Juste, Ana Hurtado

**Affiliations:** Department of Animal Health, NEIKER—Instituto Vasco de Investigación y Desarrollo Agrario, Berreaga 1, Bizkaia, 48160 Derio, Spain

## Abstract

Minimum inhibitory concentrations (MIC) of 13 antimicrobial agents were determined by broth microdilution for 72 *Campylobacter jejuni* strains from livestock. Twenty-three (31.9%) isolates were fully susceptible; all isolates were susceptible to erythromycin, chloramphenicol, streptomycin, gentamicin, sulfamethoxazole, and meropenem, and all but one to kanamycin. Resistance to quinolones was highest (52.8%), reaching similar values among poultry, dairy cattle, and sheep, but lower in beef cattle. Resistance to tetracyclines (48.6%) was mainly associated to dairy cattle and *β*-lactams (26.4%) to poultry. Multidrug resistance was mainly detected in dairy cattle (28.6%) and poultry (21.0%), whereas beef cattle had the highest percentage of fully susceptible isolates. Two real-time PCR assays to detect point mutations associated to quinolone (C257T in the *gyr*A gene) and macrolide (A2075G in the 23S rRNA genes) resistance were developed and validated on these strains. The analysis of a further set of 88 isolates by real-time PCR confirmed the absence of macrolide resistance and demonstrated the reproducibility and processability of the assay.

## 1. Introduction

Campylobacters are asymptomatically present in the intestinal tract of a wide range of mammals and birds, including domestic farm animals, which constitute potential sources for human infection via contamination of food and water. The incidence of *Campylobacter jejuni* has increased during the last decade, and today it is the leading cause of bacterial enteritis in most developed countries [1, 2]. Although the majority of *Campylobacter* infections are self-limiting, complicated cases may warrant antimicrobial therapy. Antimicrobial susceptibility data show an increase in the number of fluoroquinolone-resistant and, to a lesser extent, macrolide-resistant *Campylobacter* strains causing human infections. Resistance problems in human medicine are mainly linked to human misuse of antimicrobial agents, but there is accumulating evidence that antimicrobial resistance originating from the use of antimicrobials in food animals might complicate therapy of human infections [3, 4]. Antimicrobials used therapeutically or prophylactically in animal husbandry can also exert selective pressure on bacteria that infect food animals and reach humans via food products [5]. Local and international committees have highlighted the need for better control of antibiotics usage in human medicine and veterinary husbandry [2]. In this sense, systematic monitoring of the occurrence of antimicrobial resistance in *C. jejuni* originating from animals can serve as an indicator of the selective pressure these bacteria are undergoing and help in early detection of resistance development. 

 A prevalence study recently carried out in the Basque Country (Northern Spain) identified 28.3% (34/120) of ovine and 18.0% (37/206) of bovine farms positive for *C. jejuni *[6], and even higher values (38.2%, 13/34) in free-range poultry farms [7]. The high incidence of human *Campylobacter *infections in the Basque Country (114 cases per 100,000 inhabitants in 2006) and the relatively high prevalence in primary production units of this foodborne pathogen prompted us to determine the antimicrobial resistance profiles of a selection of *C. jejuni* isolated from animal faeces in the Basque Country over two years [6, 7].

## 2. Materials and Methods

### 2.1. Selection of Isolates

*C. jejuni* isolates were selected from a prevalence study of thermophilic campylobacters in livestock carried out in the Basque Country (Northern Spain) between October 2003 and May 2005 [6, 7]. To avoid bias, isolates were selected on the basis of isolation source (host, farm, and flock). Hence, the 72 isolates analysed by broth microdilution included 19 isolates from 12 poultry farms (18 flocks), 25 from dairy sheep (21 farms), and 28 isolates from cattle (14 beef cattle and 11 dairy cattle farms). When isolates were related epidemiologically, that is, originating from the same flock/herd, previous *fla*A PCR-RFLP and PFGE typing data [6, 7] were considered to select genetically unrelated strains. Thus, the 72 isolates could be discriminated into 50 PFGE and 28 *fla*A PCR-RFLP types. With the exception of isolates from beef cattle, some of the additional 88 isolates analysed by real-time PCR were in some manner related to the initial set of 72 isolates (isolated from animals in the same herd/flock) and therefore not used to estimate prevalence rates of resistance. 

*C. jejuni* strains from the CAMPYNET collection (strain CNET-015 susceptible to both macrolides and quinolones; strain CNET-077 resistant to quinolones) and *Campylobacter coli* strain 0402587 (resistant to macrolides) were used as controls for SNP detection by real-time PCR.

### 2.2. Broth Microdilution Test

Minimum inhibitory concentrations (MIC) were determined for 72 strains isolated from poultry (19), dairy sheep (25), and cattle (28) by broth microdilution using a custom-designed Susceptibility Plate (Sensititre, Trek Diagnostic Systems) containing serial two-fold dilutions of 13 antimicrobial agents (Table 1). *C. jejuni* inocula for MIC were prepared from overnight growth on Columbia agar incubated in microaerobic atmosphere (10% CO_2_, 5% O_2_, 85% N_2_) at 41.5 ± 1°C by suspension in 5 mL of Sensititre Standardization Broth (turbidity equivalent to 0.5 McFarland standard). One hundred microlitres of the suspension were transferred into 11 mL of cation-adjusted Mueller-Hinton supplemented with 5% lysed horse blood, and 100 *μ* *L* were then used to inoculate the 96-well panel to give a final concentration of 10^5^ cfu/mL. After microaerobic incubation for 24 hours at 41.5 ± 1°C, growth in each well was compared with that of the positive control (well with no antimicrobial drug) and MICs recorded as the lowest concentration of the antimicrobial that completely inhibited growth, except for sulfamethoxazole, for which MICs were set to the lowest concentration that inhibited 80% of growth. Results were interpreted using epidemiological cut-off values based on the distribution of MICs of wild type susceptible populations as developed by the European Committee for Antimicrobial Susceptibility Testing (EUCAST, http://www.eucast.org), except for kanamycin, meropenem, and sulfamethoxazole, where CLSI breakpoints between susceptible and intermediate for Enterobacteriaceae strains were used [8] as indicated in Table 1.

### 2.3. SNP Discrimination by Real-Time PCR

Two TaqMan real-time PCR assays were developed to detect point mutations associated to quinolone (C257T in the *gyr*A gene, Thr-86-Ile) and macrolide (A2075G mutation in the 23S rRNA genes) resistance. Different *Minor Grove Binding* (MGB) and **Locked Nucleic Acid** (LNA) probes were tested, and those combinations providing the best mismatch discrimination (based on net fluorescent difference between maximal and minimal fluorescent signals and the beginning of the exponential growth phase of the reaction) were selected. Ten *μ*L reactions included 900 nM of each primer, 100–200 nM of probe (Table 2), 1 × TaqMan Universal Master Mix (Applied Biosystems) and Ampli-Taq Gold DNA polymerase, and 5 ng of template DNA. PCR reactions were run on an ABI Prism 7500 Sequence Detection System (Applied Biosystems) using the following program: 10 minutes at 95°C, and 40 cycles of 15 s at 95°C, and 1 minute at 60°C. SNP discrimination by real-time PCR was initially performed on the 72 isolates analysed phenotypically and then used to genotypically characterise a further set of 88 isolates, adding up to a total of 160 isolates distributed as follows: 36 poultry isolates from 13 farms (21 flocks), 44 ovine isolates from 35 farms, and 80 cattle isolates from 38 farms.

### 2.4. Statistical Analyses

Comparison of frequencies of resistance/susceptibility among host species was carried out using Fisher exact test, and quantitative MIC values were transformed into a base 2 logarithm to reduce variability and to have a two-fold scale for the final units, and then submitted to analysis of variance and comparison of least square means in a factorial design for antibiotic and host species using the GLM procedure on the SAS statistical package version 8.0 (SAS Institute, USA). *P*-values less than .05 were considered significant.

## 3. Results

Distribution of MICs for the 72 *C. jejuni* strains analysed by broth microdilution are shown in Table 1. Twenty-three (31.9%) isolates were susceptible to all the 13 antimicrobial agents. In addition, all isolates were susceptible to erythromycin (MICs not higher than 0.5 mg/L), chloramphenicol, streptomycin, gentamicin, sulfamethoxazole, and meropenem, and most of them (83.3%) had MIC values of 2–4 mg/L for kanamycin, with only one isolate from dairy cattle resistant. Resistance to quinolones and tetracyclines was the most common trait. Hence, 38 isolates (52.8%) were resistant to both ciprofloxacin and nalidixic acid, while the remaining 34 (47.2%) were susceptible to both (MIC ≤ 0.25 and 8, respectively). Resistance to tetracycline was observed for 35 (48.6%) isolates, with MIC ≥ 16 mg/L for 30 of them. All but one of the isolates resistant to tetracycline were also resistant to doxycycline, the one exception being an ovine isolate with an MIC value of 8 for tetracycline and 0.5 for doxycycline. Nineteen (26.4%) isolates were resistant to ampicillin and amoxicillin. Multidrug resistance defined as resistance to three or more classes of antimicrobial agents, was present in 10 *C. jejuni* isolates (Table 3), mainly associated to dairy cattle (28.6%), closely followed by poultry (21.0%). Another 23 isolates were resistant to two classes of antibiotics, most of them to quinolones and tetracyclines. 

**Figure 1:**
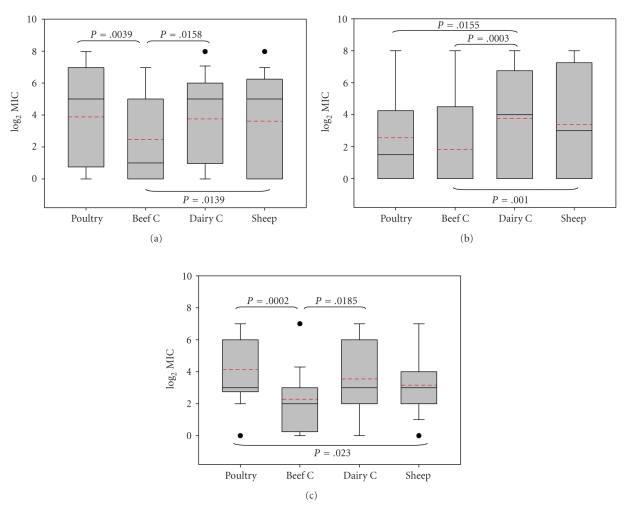
Box Plots representing log_2_-transformed MIC values distribution among host species for (a) Quinolones, (b) Tetracyclines, (c) *β*-lactams. To standardise MIC values for all antimicrobials, MICs were divided by the lowest value tested for each antimicrobial before log transformation. The boundary of the box closest to zero indicates the 25th percentile; the continuous line within the box marks the median; a dashed line marks the mean and the boundary of the box farthest from zero indicates the 75th percentile. Error bars above and below the box indicate the 90th and 10th percentiles. Outlying points are represented as closed dots. Significant differences (*P* < .05) are indicated with their corresponding *P* values.

 It was interesting to note that the overall resistance prevalence found for isolates from beef cattle was lower compared to sheep, dairy cattle, and poultry. Hence, beef cattle were the host species with the highest percentage of isolates sensitive to all 13 tested antimicrobial agents (50.0%), whereas sheep had the lowest (24.0%) (Table 3). The number of isolates tested phenotypically was too small to provide any statistical resolution when comparing the frequencies of resistance/susceptibility among host species. However, comparison of MIC values showed a different distribution for isolates from different host species (Figure 1). Analysis of variance and comparison of log-transformed mean MIC values detected significantly lower values for quinolones among *C. jejuni* from beef cattle compared to all other three host species (*P* < .05) which did not differ among themselves (Figure 1(a)). Similarly, values for tetracyclines and *β*-lactams also differed between species (Figures 1(b) and 1(c)). 

 Since fluoroquinolones and macrolides are the drugs of choice for the treatment of *Campylobacter* infections, two real-time PCR assays were developed to rapidly determine the distribution of genetic determinants associated to resistance to these antimicrobials: point mutations in the *gyr*A gene encoding the GyrA subunit of the DNA gyrase associated to quinolone resistance (C257T in the *gyr*A gene, Thr-86-Ile) and in the domain V of the 23S rRNA gene (A2075G mutation) that mediates resistance to macrolides. SNP discrimination by real-time PCR was initially performed on the 72 *C. jejuni* strains phenotypically analysed by broth microdilution. When compared with phenotypic results, real-time PCR detected the *gyr*A gene C → T nucleotide point mutation in all the strains identified as resistant to ciprofloxacin and nalidixic acid by broth microdilution, but none of the susceptible ones. Also in fully agreement with the phenotypic antimicrobial sensitivity test, none of the strains had the A2075G mutation in the 23S rRNA genes that confers resistance to macrolides, as shown by real-time PCR. Once established the 100% concordance between broth microdilution and real-time PCR, a further set of 88 isolates was then genotypically analysed by real-time PCR. Again, all of them were susceptible to macrolides but resistance to quinolones was observed. Overall, 89 of 160 isolates had the quinolone-resistant mutation in the *gyr*A gene, with a similar distribution among different host to that observed by broth microdilution. Comparison of frequencies of resistance/susceptibility to quinolones as determined by real-time PCR among different hosts showed significantly lower resistance rates in beef cattle than in the other species (*P* < .05).

## 4. Discussion

The use of different antimicrobial susceptibility testing methods, antimicrobial panels, and breakpoints hampers comparison of antimicrobial resistance distribution data between studies. Agar dilution is the method most often recommended for *Campylobacter* spp. (CLSI), but it is labour-intensive and difficult to perform in routine laboratories. Broth microdilution is a fast and easy-to-perform method that yields reproducible MIC results for *C. jejuni *[9, 10], it is commercially available, and the standardised method was approved by CLSI in 2006 [11]. Antimicrobials and dilution ranges used in the study herein included those defined by the EU as a minimum requirement (tetracycline, erythromycin, ciprofloxacin, gentamicin and streptomycin) and the additional recommendations (ampicillin, amoxicillin and nalidixic acid) [12], along with others commonly used in clinical practice or in animal productions as such or their derivatives (Table 1). Since the purpose of this study was to monitor for antimicrobial resistance surveillance and detect the development of microbiological resistance, MIC epidemiological cut-off values rather than clinical breakpoints were used, which in some cases resulted in a higher proportion of isolates categorized as resistant. When compared with the 2006 European Community report on trends and sources of zoonotic agents and antimicrobial resistance [2] using the same cut-offs, our results showed a high proportion of poultry strains resistant to ciprofloxacine, above the estimates reported by most countries. Resistance to tetracycline was within the ranges estimated for several Member States. These, along with the absence of erythromycin, streptomycin. and gentamicin resistance represent a situation similar to that reported by the Netherlands or Germany, but significantly different to Spain, that reported much higher resistance levels to all these antimicrobials. Also in broilers, in a survey carried out from 1997 to 1998 in slaughterhouses in a nearby region in Spain (La Rioja) using agar disk diffusion [13], approximately 99% of *C. jejuni *isolates were fluoroquinolone resistant; resistance to gentamicin was also higher than in the present study, but comparable for tetracycline and ampicillin. The poultry strains analysed in the study herein had been isolated from free-range chicken farms [7] where antibiotics are rarely used and entirely confined to therapeutic treatment of diseased animals, whereas in the abovementioned studies strains originated from broilers. Although the results of studies comparing the antimicrobial susceptibility patterns of *Campylobacter* recovered from poultry raised in different production systems (conventionally reared *versus* free-range chickens) are somewhat inconclusive, it does appear that fluoroquinolone resistance is more frequently reported in isolates from conventionally reared poultry [14]. However, recent studies suggest that fluoroquinolone-resistant *Campylobacter,* once evolved, may continue to persist in chicken flocks regardless of the use of fluoroquinolones. Luo et al. [15] examined the effect of a resistance-conferring C257T mutation in the *gyr*A gene on the fitness of fluoroquinolone-resistant *C. jejuni* in chicken. Their results demonstrated enhanced in vivo fitness of fluoroquinolone-resistant *C. jejuni* in chicken in the absence of antibiotic selection pressure, suggesting that fluoroquinolone-resistant isolates may be able to persist in the chicken reservoirs even without the usage of fluoroquinolone antimicrobials. Epidemiological studies also showed the persistence of fluoroquinolone-resistant *C. jejuni* in chicken flocks after ceasing on-farm fluoroquinolone use [16]. In this study, all the fluoroquinolone-resistant isolates had the C257T mutation in the *gyr*A gene. 

 In dairy cattle, resistance was higher to tetracyclines followed by fluoroquinolones, a similar pattern to that found by Englen et al. [17] in US dairy cattle despite differences in the method and breakpoints used. Contrary to our results, the same authors [18] had reported similar levels of tetracycline resistance in feedlot cattle. In Europe, resistance is highly variable from country to country [19, 20], with either tetracyclines or quinolones as the antimicrobials towards resistance are higher among *C. jejuni* isolated from beef cattle. Macrolide resistance, absent in this study, was reported albeit at low levels in USA and Europe [17–19]. Despite the high prevalence of *C. jejuni* in sheep, few investigations on antimicrobial resistance have been conducted in ovine isolates and comparable data is scarcely available. Zweifel et al. [21] found very low rates of resistance in Swiss ovine campylobacters using disk diffusion and the NCCLS standards. In the present study, along with quinolones, resistance was also high to tetracyclines. Interestingly, a recent study reported the emergence of a tetracycline-resistant *C. jejuni* clone associated with outbreaks of ovine abortion in the USA [22]. In our region, *Campylobacter* infection is not among the main causes of ovine abortion [23]. 

 The developed *gyr*A and 23S rRNA genes SNP detection assays by real-time PCR were able to detect the correct genotype in all cases providing a rapid and reproducible screening method for quinolone and macrolide resistance detection, which allowed the analysis of a larger set of strains. Other molecular methods commonly used to detect the point mutation at the 86-codon of the *gyr*A gene are mismatch amplification mutation assay PCR (MAMA-PCR) [24], single-strand conformation polymorphism analysis [25], the latter having the advantage of detecting mutations at neighbouring positions, but both difficult to standardize or automate. Real-time PCR has also been used, either using TaqMan probes [26] or by means of melting peak analysis [27], also used to detect the A2075G mutation in the 23S rRNA genes associated to macrolide resistance in *Campylobacter *spp. [28]. The real-time PCR designs presented herein for *gyr*A and 23S rRNA genes SNP detection take advantage of probe modifications that provide increased thermal stability allowing shorter probes to be used, and therefore, conferring increased sequence-specificity compared to ordinary DNA probes, that is, TaqMan probes conjugated with MGB ligands at the 3′ end and probes with LNA nucleotides at different positions. This characteristic allowed us to design probes that avoid the polymorphic site described by Wilson el al. [26] in the third position of their *gyr*A probe, thus obtaining better allelic discrimination. Also important are other possible mutations within the sequences recognised by the probes described for both the *gyr*A gene (A256G, associated to nalidixic acid resistance, but not ciprofloxacin resistance) and the 23S rRNA gene (A2058C, associated to higher erythromycin MICs) of a few *C. jejuni* isolates [29, 30]. These mutations would most likely produce a negative or inconclusive result by real-time PCR and would require sequencing analysis. Nevertheless, this was never the case in the study herein, where cross-resistance between nalidixic acid and ciprofloxacin was found in all isolates, in agreement to other studies in Spain [13, 31], and erythromycin MICs were low. Here, real-time PCR detected the correct genotype in all cases providing a rapid and reproducible screening method for quinolone and macrolide resistance detection, which allowed the analysis of a larger set of strains. Thus, whereas the number of isolates tested phenotypically was too small to provide any statistical resolution, significantly lower quinolone resistance rates in beef cattle than in the other species could be observed when analysing results obtained by real-time PCR. 

 Resistance to fluoroquinolones has increased over the past years in many parts of the world [1–3]. In Spain, fluoroquinolone-resistant *C. jejuni* isolated from humans were first reported in 1988 [32] and resistance prevalence has increased since then to high levels [13, 31, 33]. The activity of erythromycin against *C. jejuni* human isolates, the antibiotic of choice for the treatment of diarrhea caused by *Campylobacter *strains (especially in infants), seems to remain stable at rates below 5% [13, 31]. This stable macrolide activity is in agreement with the absence of resistance among animal isolates observed in this study. This susceptibility is reassuring but active and more extensive antimicrobial surveillance in campylobacters from animals is needed to allow future informed decisions about how macrolide antibiotics could be used in food animals while still safeguarding human health. The real-time SNP discrimination protocols presented herein provide the processability required for such purposes. The analysis of large number of isolates, performed in combination with MIC determination on a smaller selection, should help in early detection of resistance development.

##  Conflict of Interest Statement

None of the authors have any conflict of interests.

## Figures and Tables

**Table 1:** Resistance (percent) and distribution of MIC for the 72 C.* jejuni* isolates. White fields denote range of dilutions tested for each antimicrobial agent. MICs above the range are given as the concentration closest to the range. MICs equal to or lower than the lowest concentration tested are given as the lowest tested concentration. Bold vertical lines indicate EUCAST epidemiological cut-off values.

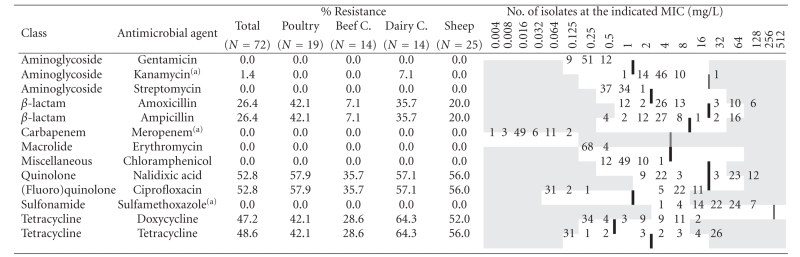

^(*a*)^No cut-off value given by EUCAST; CLSI breakpoints between susceptible and intermediate Enterobacteriaceae strains, as indicated by double vertical lines, were used.

**Table 2:** Primers and probes sequences.

Target	Name	Sequences (5′ → 3′)	C (nM)
*gyr*A	gyr-Fw	GGGTGCTGTTATAGGTCGTTATCA	900
	gyr-Rv	TTGAGCCATTCTAACCAAAGCAT	900
	Probe-gyr-S	HEX – CAT[+G]GAGAT[+A][+C][+A]GC[+A]GTTT – BHQ1	150
	Probe-gyr-R	FAM – CATGGAGATATAGCAGTTT – MGB	150

23S	23S-Fw	CAGTGAAATTGTAGTGGAGGTGAAA	900
	23S-Rv	TTCTTATCCAAATAGCAGTGTCAAGCT	900
	Probe-23S-S	HEX – CGGGGTC[+T][+T][+T]CCGTCTTG – BHQ1	100
	Probe-23S-R	FAM – CGGGGTC[+T][+C][+T]CCGTCTTG – BHQ1	200

LNA nucleotides are indicated by a + symbol and in brackets; SNPs are underlined.

**Table 3:** Resistance phenotypes of *C. jejuni *isolates by source.

No. of resistances	Resistance profile^(a)^	Poultry (*n* = 19)		Sheep (*n* = 25)		Beef cattle (*n* = 14)		Dairy cattle (*n* = 14)		Total (*n* = 72)	
		*n*	(%)	*n*	(%)	*n*	(%)	*n*	(%)	*n*	(%)
7	CpNxTDxAmAxK	0	(0.0)	0	(0.0)	0	(0.0)	1	(7.1)	1	(1.4)
6	CpNxTDxAmAx	4	(21.0)	1	(4.0)	1	(7.1)	3	(21.4)	9	(12.5)
4	CpNxTDx	4	(21.0)	9	(36.0)	1	(7.1)	3	(21)	17	(23.6)
4	CpNxAmAx	2	(10.5)	2	(8.0)	0	(0.0)	0	(0.0)	4	(5.6)
4	TDxAmAx	0	(0.0)	0	(0.0)	0	(0.0)	1	(7.1)	1	(1.4)
3	TAmAx	0	(0.0)	1	(4.0)	0	(0.0)	0	(0.0)	1	(1.4)
2	CpNx	1	(5.3)	2	(8.0)	3	(21.4)	1	(7.1)	7	(9.7)
2	TDx	0	(0.0)	3	(12.0)	2	(14.3)	1	(7.1)	6	(8.3)
2	AmAx	2	(10.5)	1	(4.0)	0	(0.0)	0	(0.0)	3	(4.2)
0	*Susceptible*	6	(31.6)	6	(24.0)	7	(50.0)	4	(28.6)	23	(31.9)

^(a)^Ax, Amoxicillin; Am, Ampicillin; Cp, Ciprofloxacin; Dx, Doxicycline; K, Kanamycin; Nx, Nalidixic acid; T, Tetracycline
